# Counterfactual time series analysis of short-term change in air pollution following the COVID-19 state of emergency in the United States

**DOI:** 10.1038/s41598-021-02776-0

**Published:** 2021-12-07

**Authors:** Tanujit Dey, Pooja Tyagi, M. Benjamin Sabath, Leila Kamareddine, Lucas Henneman, Danielle Braun, Francesca Dominici

**Affiliations:** 1 Center for Surgery and Public Health, Department of Surgery, Brigham and Women’s Hospital, Harvard Medical School, Boston, USA; 2 Department of Biostatistics, Harvard T.H. Chan School of Public Health, 677 Huntington Ave, Boston, MA 02115 USA; 3 Faculty of Arts and Sciences, Research Computing, Harvard University, 38 Oxford Street, Cambridge, MA 02138 USA; 4 Department of Civil, Environmental, and Infrastructure Engineering, George Mason University, 4400 University Drive, Fairfax, VA 22030 USA; 5 Department of Data Science, Dana-Farber Cancer Institute, 450 Brookline Ave, Boston, MA 02215 USA

**Keywords:** Environmental impact, Atmospheric science

## Abstract

Lockdown measures implemented in response to the COVID-19 pandemic produced sudden behavioral changes. We implement counterfactual time series analysis based on seasonal autoregressive integrated moving average models (SARIMA), to examine the extent of air pollution reduction attained following state-level emergency declarations. We also investigate whether these reductions occurred everywhere in the US, and the local factors (geography, population density, and sources of emission) that drove them. Following state-level emergency declarations, we found evidence of a statistically significant decrease in nitrogen dioxide (NO_2_) levels in 34 of the 36 states and in fine particulate matter (PM_2.5_) levels in 16 of the 48 states that were investigated. The lockdown produced a decrease of up to 3.4 µg/m^3^ in PM_2.5_ (observed in California) with range (− 2.3, 3.4) and up to 11.6 ppb in NO_2_ (observed in Nevada) with range (− 0.6, 11.6). The state of emergency was declared at different dates for different states, therefore the period "before" the state of emergency in our analysis ranged from 8 to 10 weeks and the corresponding "after" period ranged from 8 to 6 weeks. These changes in PM_2.5_ and NO_2_ represent a substantial fraction of the annual mean National Ambient Air Quality Standards (NAAQS) of 12 µg/m^3^ and 53 ppb, respectively. As expected, we also found evidence that states with a higher percentage of mobile source emissions (obtained from 2014) experienced a greater decline in NO_2_ levels after the lockdown. Although the socioeconomic restrictions are not sustainable, our results provide a benchmark to estimate the extent of achievable air pollution reductions. Identification of factors contributing to pollutant reduction can help guide state-level policies to sustainably reduce air pollution.

## Introduction

There is consistent evidence that short- and long-term exposure to fine particulate matter (PM_2.5_) and nitrogen dioxide (NO_2_) increases the risk of mortality, hospitalization, and other adverse health outcomes^1–6,11,12^. Furthermore, several studies have provided preliminary evidence that short and long-term air pollution exposure increases the risk of hospitalization and death among individuals with COVID-19^4–10^.

The United States mitigates air pollution through a combination of federal, state, and local air pollution regulations^13^. For example, the federal government sets emissions standards and the NAAQS. They also require states to prepare State Implementation Plans (SIPs) that detail emissions reductions strategies for areas that are not in compliance with the NAAQS (non-attainment areas). SIPs use air quality models to demonstrate how regulating local emissions sources helps a non-attainment area meet the NAAQS. Geographically heterogeneous regulations, emission sources, and meteorology, results in varying air pollution concentrations by geographic location^13,14^.

Several studies have examined the impact of a sudden intervention on changes in air pollution (see^15^ for a review). For example, researchers used interrupted time-series designs to quantify the impact of the 1990 Dublin coal ban^16^ and regression discontinuity to identify the arbitrary spatial impact of the China Huai River Policy^17^. An important feature of these studies is that they investigated abrupt and localized changes across a relatively short time span (Dublin coal ban) and spatial scale (Huai River policy)^18^. Because of the abrupt nature of these interventions, defining a hypothetical experiment in these studies was straightforward.

Similarly, we examined the effect of the abrupt lockdown measures implemented in response to the COVID-19 pandemic, which produced sudden and significant changes in how society functions, with decreases in road traffic, air traffic, and economic activity^19^. This provided us with an unprecedented opportunity to implement a quasi-experimental design with a well-defined control condition (no pandemic) to estimate the changes in air pollution because of the implementation of these extreme measures. In a quasi-experimental design, the researcher compares outcomes between a treatment group and a control group, just as in a classical experiment; but treatment status (in our context the COVID-19 related intervention) is determined by politics, an accident, a regulatory action, or some other action beyond the researcher’s control (in our context the start of the pandemic). See^20^ for a discussion of strengths and limitations of a quasi-experimental design. Furthermore, the spatial heterogeneity in the extent to which air pollution levels changed because of the lockdown measures allowed us to identify factors contributing to these changes.

A number of recent studies have investigated the effect of the COVID-19 pandemic on the levels of different air pollutants in the US^21–34^, globally^35–39^ and for several cities around the world^40–51^. Table 1 summarizes studies that have estimated changes in air pollution levels by comparing air pollution levels during the COVID-19 pandemic period to historical data both in the US and globally.

**Table 1:** Summary of published studies examining changes in air pollution attributable to COVID-19 related interventions in the US and globally.

Citation	Geographic locations	COVID19 related intervention	Confounding adjustment	Statistical approach	Results
Berman et al.^22^	United States (all counties in the U.S. with both NO_2_ and PM_2.5_ monitors)	Reduced traffic and mandated business closures between March 13-April 21. March 13th being when U.S. reported cases exceeded 2000 and the first enacted state-wide social distancing order	None	Two-sided t-tests paired by county (α = 0.05)	25.5% reduction (4.8 ppb) in NO_**2**_ was observed during the COVID-19 period
					NO_2_ decline was statistically significant regardless of when mandated business closures were implemented
					11.3% statistically significant reduction (0.7 μg/m^3^) of PM_2.5_ in counties from states that instituted early non-essential business closures
Gillingham et al. ^28^ (Commentary)	United States (785 monitors)	Shutdowns	Weather and seasonality	Global polynomial and a two-step local regression	PM_2.5_ concentrations have decreased by around -0.5 μg/m^3^ since the start of the shutdowns
					Estimated 11% NOx decrease in daily local emissions
					There is insufficient evidence to prove that there was a significant decrease in PM_2.5_ concentrations in the U.S
Goldberg et al.^24^	20 cities in North America	COVID-19 Physical distancing measures (lockdown) (15 March to 30 April post-covid-19 period)	Solar zenith angle and meteorological conditions over very short time scales	Average differences	Adjusted for seasonality and meteorology, NO_2_ had a median drop of 21.6% before and after COVID‐19 physical distancing
Karaer et al. ^25^	Florida	COVID-19 social distancing behaviors (March 2020)	Population density and income	A cross-correlation based dependency analysis	The decrease in NO_2_ concentrations and vehicle miles travelled (VMT) started 2 weeks before the official stay-at-home order and resulted in 54.07% and 59.68% decrease in NO_2_ and VMT by the end of the month, respectively
Miech et al. ^27^	Phoenix	COVID-19 Stay at home orders (pre-COVID-19: Jan 6-March 6 & Post-COVID-19: March 13-April 8)	Meteorological parameters (horizontal wind speed, temperature, precipitation, and planetary boundary layer height)	Linear regression model	No uniform decrease was found in CO or NO_2_ across the three sites studied
					There was a significant decrease (45%) in PM_10_ at all the sites compared to the past two years
Parker et al. ^26^	Southern California	Stay-At-Home orders (19 March-30 June of the last 5 years)	Meteorological differences	Average differences	Concentrations of PM_2.5_ and NO_x_ showed an overall reduction (10–45% and 13–40%, respectively) across the basin in 2020
					O_3_ concentrations decreased (9 ppb or 22%) in the western part of the basin and increased (8 ppb or 15%) in the downwind areas
Venter et al. ^36^	34 countries	Lockdown (Jan 1- May 15)	Meteorological variability	Linear regression models	11 μg/m^3^ reduction in NO_2_ (on average 60% reduction)
					12 μg/m^3^ reduction in PM_2.5_ (on average 31% reduction)
					4 μg/m3a increase in O_3_ (4% increase)
Fu et al.^35^	20 selected major cities around the world	Lockdown (lockdown period in each city compared to same period in the past 3 years)	Meteorological variability	ANOVA and Tukey’s HSD tests	NO_2_ decreased significantly in all cities relative to the past 3 years
					PM_2.5_ decreased in all cities and found a significant decrease in 9 cities relative to each of the 3 years
Benchrif et al. ^50^	21 selected cities around the world	Lockdown	None	Descriptive statistics	PM25 and NO2 concentrations declined considerably in different cities during lockdown period
Hammer et al.^51^	China, Europe, and North America	Lockdown (Jan – Apr 2020)	None	Descriptive statistics and simulation study	PM_2.5_ concentrations decreased in all study locations compared to same period during 2018 and 2019

Regardless of the emerging literature on this topic, these studies for the most part do not simultaneously account for autocorrelation, time trends and seasonality, and meteorological factors. To our knowledge, none of these studies attempt to identify state-level factors contributing to heterogeneity in the air pollution declines across states for both PM_2.5_ and NO_2_.

In this study, we had several scientific objectives that distinguish this paper from existing contributions in the literature. More specifically, we 1) develop and implement state-of-the-art time series approaches for counterfactual forecasting to predict weekly state-levels of PM_2.5_ and NO_2_ from January 1, 2020, to April 23, 2020, under the hypothetical scenario that the pandemic did not occur. These models account for measured confounding (e.g. meteorological factors), unmeasured confounding (e.g. seasonal variation and time trends) and residual autocorrelation; 2) properly validate the accuracy of the model fitting and account for the uncertainty in the counterfactual forecasts via bootstrap; 3) estimate the weekly state-level deviations and 95% CI between counterfactual (e.g., absent the pandemic) and observed levels of PM_2.5_ and NO_2_ from January 1, 2020 to April 23, 2020; 4) assess whether the deviations between the counterfactual values and the observed values start to deviate in correspondence to key interventions implemented as a result of the pandemic; 5) assess within each state, changes in both PM_2.5_ and NO_2_; and finally 6) investigate which state-level characteristics, including emissions sources, contributed the most to these changes, while adjusting for geography and population density.

## Materials and methods

### Data acquisition

We gathered and harmonized data from several databases (Table S1). We obtained historical daily monitor data of PM_2.5_ and NO_2_ concentrations for January 1, 2015 to August 31, 2019 from the US EPA Air Quality System^52^. We obtained current levels of these air pollutants for August 31, 2019 to April 23, 2020 from the EPA AirNow application programming interface^53^. We linked historical and current monitor data within each state. These data were available for 48 states for PM_2.5_ and 36 states for NO_2_. We obtained daily temperature, humidity, and precipitation data from the University of Idaho’s GRIDMET project, which were then aggregated to the state level using Google Earth Engine^54^.

We obtained state-level source emissions totals from the National Emissions Inventory for 2014^55^, and gathered information on population density and geographic region classification of the states from the United States Census Bureau^56,57^. Finally, we accessed the COVID-19 US State Policy Database^58^ to extract information regarding the dates of COVID-19 related state interventions, including state-level declaration of emergency, shelter-in-place orders, and non-essential business closures for each state. All the data sources are publicly available, they are summarized in Table S1, and also available on GitHub along with all code necessary to conduct the analysis; https://github.com/NSAPH/USA-COVID-state-level-air-pollution-SARIMA-analysis.

### Statistical methods

#### Counterfactual forecasting of air pollution levels starting January 1, 2020

SARIMA models are autoregressive models often used to forecast time series where future observations are correlated with past observations^59,60^. They have the advantage of accounting for the time trend, seasonality, confounders (e.g., meteorological variables), and residual autocorrelation. We fitted SARIMA models to historical data using weekly state-level air pollution levels (from January 1, 2015, to December 31, 2019) accounting for time trend, seasonality, autocorrelation and also accounting for the effect of weather by including temperature, precipitation, and humidity as covariates in the model.

The basis of the SARIMA model is a linear regression of a response variable Y_t_ at time t against the past values (Y_t-1_, Y_t-2_, ….) of Y and the past forecast errors (ɛ_t-1_, ɛ_t-2_, …). A detailed example of this analysis for NO_2_ in California is provided in the supplementary materials, including model validation measures (Figures S1-S5).

We conducted the following analyses separately for PM_2.5_ and NO_2_ and for each state. The algorithm of the model construction and prediction is presented below.

1. We created 1,000 time series bootstraps using Box-Cox and Loess-based decomposition^61^ to separate the time series into the trend, seasonal, and remainder part. The remainder is then bootstrapped. We used historical data from January 1, 2015, to December 31, 2019 (see Figure S2 for an example of NO_2_ in California).
2. For each bootstrapped time series, we:
  Fit SARIMA models^59–61^ adjusting for meteorological factors, namely temperature, precipitation, and humidity (see Figure S3 for an example of NO_2_ in California).
  From the fitted SARIMA models, we predict air pollution counterfactual levels (absent the pandemic) during a 16-week period from January 01, 2020, to April 23, 2020 (see Figure S4 for an example of NO_2_ in California).
3. For each state and for each week, we average the predicted air pollution counterfactual levels across all bootstrap replicates. We denote these averages by $C_{i,j}^{pred} ,\;where\; i = 1,2, \ldots ,16$, and *j* indicates the state (see Figure S4 for an example of NO_2_ in California).
4. For each state j and for each week i, we estimate the weekly differences$\delta_{i, j} = C_{i,j}^{obs} - C_{i,j}^{pred} , \;i = 1,2, \ldots ,16$, between the observed values (under pandemic conditions) and the predicted (assuming that the pandemic did not occur) (see Figure S5 for an example of NO_2_ in California). The quantification of the statistical uncertainty of these weekly differences using the bootstrap replicates is called “bagged SARIMA” (see Figures S4 and S5 for an example of NO_2_ in California).

The data and code for the analysis is available at https://github.com/NSAPH/USA-COVID-state-level-air-pollution-SARIMA-analysis.

#### Model assessment

To assess the overall predictive performance of the SARIMA model, we repeated the same procedure of model building and prediction as described in the algorithm above, this time training the model based on the data from January 1, 2015 to December 31, 2018, and predicting for a 16-week period from January 01, 2019 to April 23, 2019. This allows us to assess model fit and evaluate our modeling approach absent the pandemic. The main goal of implementing this assessment is to find out the model’s performance in prediction absent the pandemic and compare its predictive performance using the average prediction error as defined below during the pandemic.

Average prediction error (APE) for state *j*:

$${\text{APE}}j = 1/16\sum\limits_{(i = 1)}^{16} {\delta_{(i,j)} } ,\;where\;\delta_{(i,j)} = C_{(i,j)}^{obs} - C_{(i,j)}^{pred}$$

as defined in Step 4 of algorithm above.

We used the R package auto.arima to select model coefficients with the best predictive capability based on bias-corrected Akaike Information Criterion (AIC)^62,63^ and then used the mean absolute scaled error (MASE) to evaluate the fit of the model^64^.

#### Estimating air pollution changes attributable to state-level emergency declarations

In step 2 described above, we start the counterfactual forecasting for the period January 01, 2020, to April 23, 2020 without any consideration regarding the date of the intervention (such as the declaration of the state emergency). After the forecasting was complete, we then chose the declaration of the state of emergency as the intervention because it most closely visually aligned with the onset of deviations from the forecasted pollutant concentrations. Other interventions, including the timing of non-essential business closures and shelter-in-place orders, were considered visually (see Figures S7 and S8 in the supplementary material, the differences between these interventions are less than two weeks).

We use T_int, j_ to denote the date of the state intervention (declaration of the state of emergency) for each state *j*. For each state and for each of the two pollutants (PM_2.5_ and NO_2_), we estimated the parameter $\Delta_{j}$ denoting the change in pollutant concentrations following the state intervention compared to before by calculating:

1 $$\Delta_{j} = \Delta_{before,j} - \Delta_{after,j}$$

where $\Delta_{before,j}$ is the median of the weekly deviations, ${\delta }_{i,j}$, (as defined in step 4 above) for the weeks *before* the date of the declaration of the state emergency (T_int,j_) and $\Delta_{after,j}$ is the median of these weekly deviations, ${\delta }_{i,j}$ , for the weeks *after* T_int,j_. Because of the good fit of the SARIMA model to the historical data (Figure S6), and because the counterfactual forecasting is agnostic to the date of the state level emergency (see Figures S4, S5 for an example of NO_2_ forecasting in California), we argue that negative estimated values of ${\Delta }_{j}$ indicate that air pollution levels declined because of the state-level emergency. We note that since the state of emergency was declared at different dates for different states, and the total length of the prediction period was 16 weeks in 2020, therefore the period "before" the state of emergency in our analysis ranged from 8 to 10 weeks and the corresponding "after" period ranged from 8 to 6 weeks.

To identify the states with the most pronounced discrepancy between the pattern of change in PM_2.5_ and NO_2_, we calculated the ratio (ρ_j_) for each state *j*, defined as:

2 $$\rho_{j} = \Delta_{{NO_{2} ,j}} /\Delta_{{PM_{2.5} ,j}}$$

I f ${\rho }_{j} <0$ the two pollutants changed in opposite directions (i.e., one increased while the other decreased), and the larger the magnitude of ${\rho }_{j}$ , the larger the discrepancy between the pollutants’ patterns of change.

#### Regression modeling to identify state-level factors contributing to heterogeneity in the air pollution across states

In this part of the analysis, our goal is to quantify the associations between the change in pollutant concentrations during the forecasting period January 01, 2020 to April 23, 2020 and several sources of pollutants along with a few geographical variables. The estimated ${\Delta }_{j}$ (as defined in Eq. 2) is the outcome for each state for each of the two pollutants, separately. We have used the following independent variables: the proportion of emissions from fire sources, stationary sources, and mobile sources (obtained from 2014); population density; and region of the state.Note that the NEI reports four sources of emission: fire, mobile, stationary and biogenic; we used only three of these (fire, stationary, and mobile sources) as predictors in the regression model and therefore, their proportions do not sum to 1. Instead of using a regular multivariable linear regression model, we chose to use a weighted multivariable linear regression (WMLR) model. The reasons behind using this model are: (1) this model can incorporate the covariance matrix of errors which is quite beneficial for the heteroscedastic data, which is a feature of these data sets (2) because of the variability in pollutants concentrations across states, the WMLR are more robust to the outliers than regular regression models. Lastly, based on the pairwise correlation assessments, besides the main effect of the predictors in the models, we also included all two-factor interaction terms of the predictors. This model not only quantifies better associations between the outcome and the covariates; the goodness of fit performances, using the regular R^2^ and adjusted R^2^, are better for these models with the interaction terms compared to models without the interaction terms.

## Results

### Short-term change in air pollutants following the COVID-19 state of emergency

**Figure 1:**
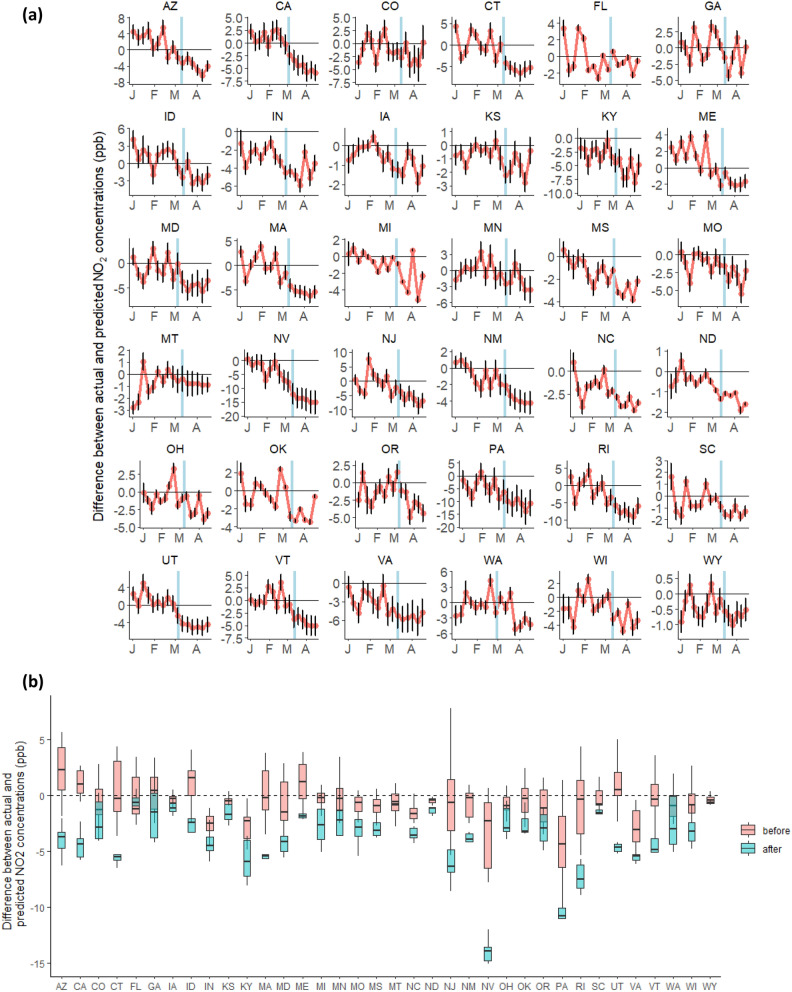
(**a**) Weekly deviations between observed NO_2_ concentrations and counterfactual predictions (e.g., absent the pandemic) for each state. The counterfactual predictions were made for 16 weeks from January 1 to April 23, 2020. The blue vertical line marks the date of the declaration of a state of emergency in each state. (**b**) Boxplots of the weekly deviations for the weeks before (pink) and for the weeks after (blue) the date of the declaration of a state of emergency in each state.

**Figure 2:**
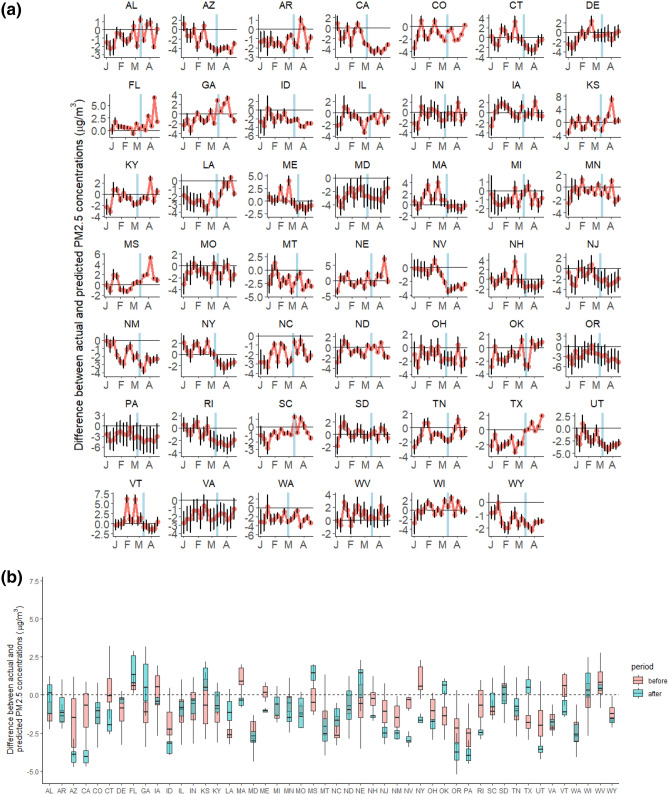
(**a**) Weekly deviations between observed PM_2.5_ concentrations and counterfactual predictions (e.g., absent the pandemic) for each state. The predictions were made for 16 weeks from January 1 to April 23, 2020. The blue vertical line marks the date of the declaration of a state of emergency in each state. (**b**) Boxplots of the weekly deviations for the weeks before (pink) and for the weeks after (blue) the date of the declaration of a state of emergency in each state.

For most states, the differences between the SARIMA counterfactual predictions (i.e., assuming the pandemic did not occur) and the observed pollutant values were close to zero during the period before the state-level emergency declaration (Figs. 1, 2), but there were significant deviations following the intervention lockdown measures.

We found evidence of a statistically significant decrease in NO_2_ concentrations following the declaration of a state of emergency in 34 of the 36 states that were investigated (Fig. 1 and Tables S2-a, S3). The change in NO_2_ following the declaration of a state of emergency ${\Delta }_{j}$ calculated using Eq. 2, ranged from -0.6 ppb to 11.6 ppb across the states, with an average change of 3.1 ppb and standard deviation 2.4 ppb.

**Figure 3:**
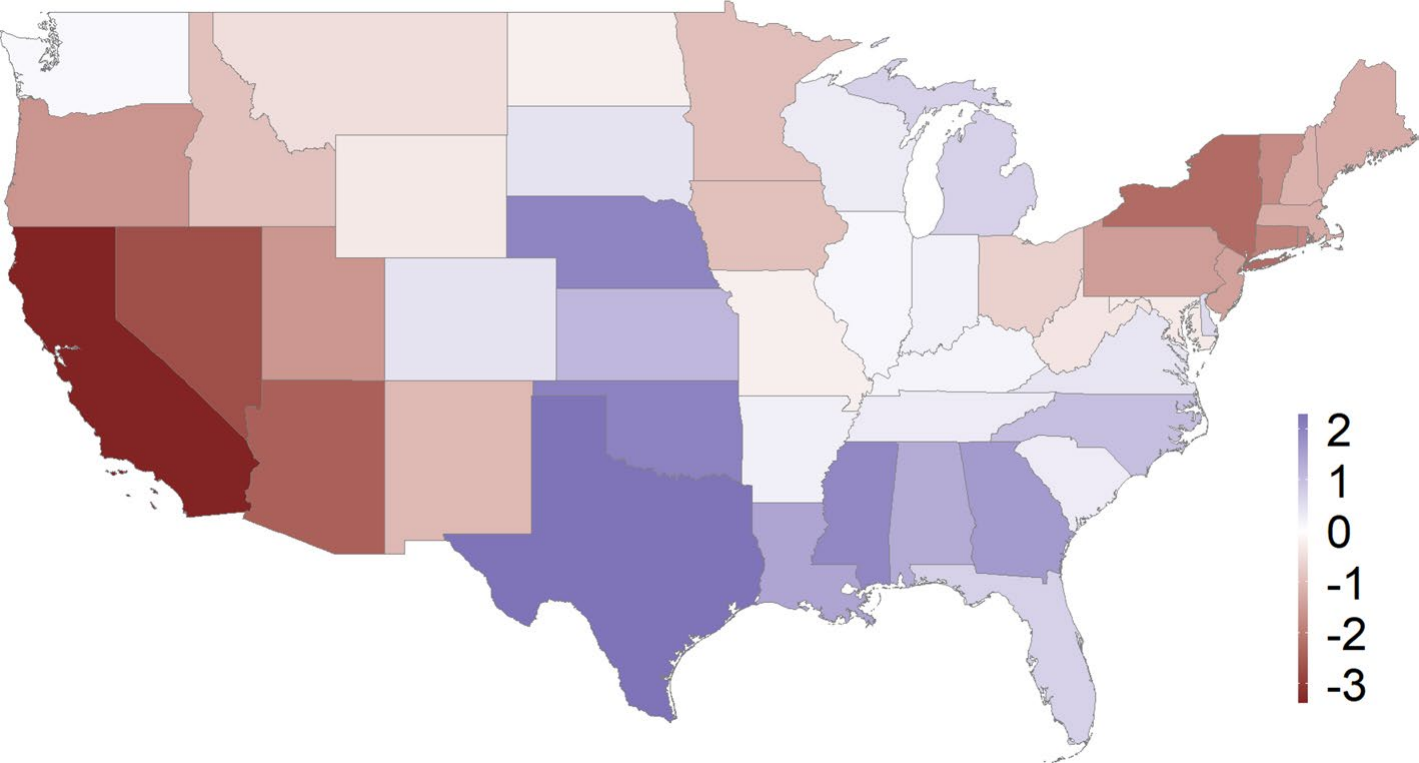
Median change in PM_2.5_ following the state-level emergency declaration for each state ($\Delta_{j}$). A negative estimated value of $\Delta_{j}$ indicates that air pollution levels declined as a result of the state-level emergency declaration. This figure was created using open source software R 4.1.0 (https://cran.r-project.org/). The base US map was used by using the R package: rnaturalearthhires (). The source code (Rcode_PM25_USmap_figure3.R) to recreate this figure, please visit our GitHub page: https://github.com/NSAPH/USA-COVID-state-level-air-pollution-SARIMA-analysis.

We also found evidence of a statistically significant decline in PM_2.5_ concentrations in 16 of the 48 states that were studied, including New York and other states in the Northeast and West Coast (Figs. 2, 3, Tables S2-b, S4, and Figure S9). The change in PM_2.5_ following the declaration of a state of emergency ranged from -2.3 µg/m^3^ to 3.4 µg/m^3^ across the states, with an average change of 0.3 µg/m^3^ and standard deviation 1.3 µg/m^3^.

In Figures S10 and S11 we show the difference between actual and predicted levels of NO_2_ and PM_2.5_, respectively for all states. Even though the date of the lockdown was not incorporated into the SARIMA model for counterfactual forecasting, the observed values were closer to the predicted values of NO_2_ before the state of emergency declarations compared to after the state of emergency declarations. For PM_2.5_ the differences before and after the state of emergency declarations are not as large.

**Figure 4:**
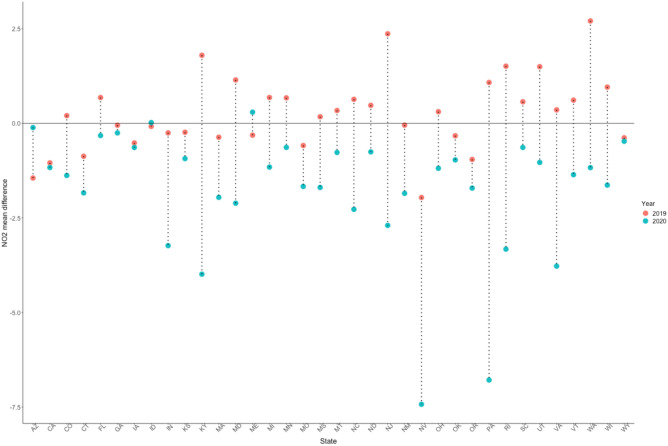
Average prediction error for each state during the same prediction period (January 1 to April 23) for 2019 (no pandemic, red) and 2020 (pandemic, blue) for the NO_2_ pollutant model (the circles are connected by the dotted line for improved visualization, no other intention is associated in this connection).

**Figure 5:**
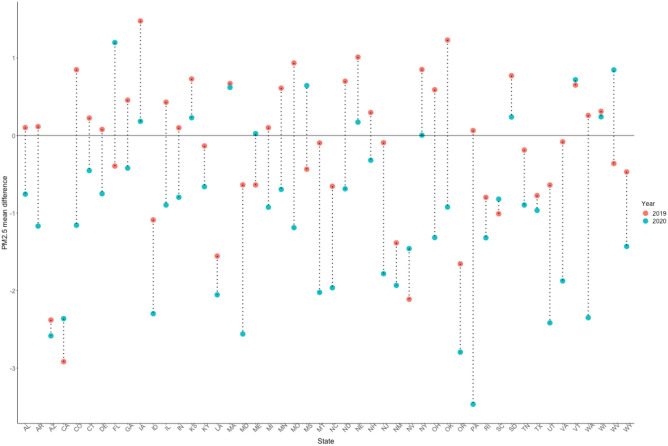
Average prediction error for each state during the same prediction period (January 1 to April 23) for 2019 (no pandemic, red) and 2020 (pandemic, blue), for the PM_2.5_ prediction model (the circles are connected by the dotted line for improved visualization, no other intention is associated in this connection).

To quantify how well the SARIMA models can predict for a given period, as described in the Methods section we compared the predictive performances of the models during the same period for 2019 (no pandemic) compared to the main analysis for 2020 (pandemic). We assessed how the APE behaves for both 2019 and 2020 for each state. From Figs. 4 and 5, except for a few states, the APEs are higher for 2020 compared to 2019 for each pollutant model. In addition to using the APE, we also summarize information regarding the model evaluation in the supplementary materials. Figure S3 shows that, for example, the SARIMA model has excellent goodness of fit for the historical data for NO_2_ in California.

We also found that MASE for the fitted models was less than 1 unit for each pollutant in each state (Figure S6), indicating that the SARIMA model outperformed one-step naïve forecasts, which use the value at time ‘t’ to predict the outcome at t + 1.

**Figure 6:**
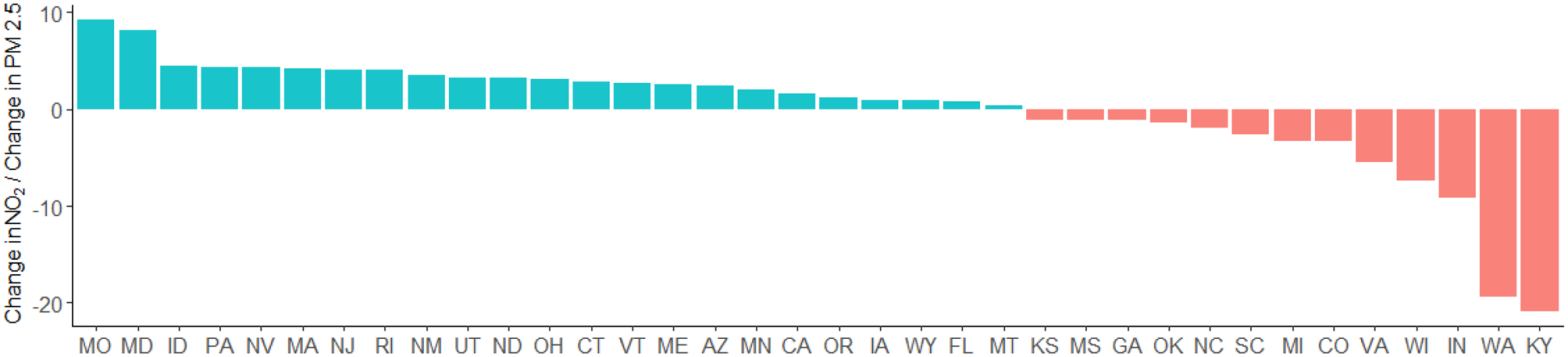
Ratio of the estimated $\Delta_{j}$ for NO_2_ divided by the estimated $\Delta_{j}$ for PM_2.5_ (ρ). A negative ratio implies that the change in NO_2_ following the declaration of the state of emergency was in the opposite direction of the corresponding changes for PM_2.5_ (i.e., one pollutant increased while the other decreased). For example, in KY, we found a decline in NO_2_ but an increase in PM_2.5_ following the state-level emergency declaration.

Figure 6 shows the estimated ρ_j_ defined as the ratio of the estimated ${\Delta }_{j}$ for NO_2_ divided by the estimated ${\Delta }_{j}$ for PM_2.5_. More than one-third of the states (13 states) had $\rho <0$ , i.e., these states experienced a decrease in NO_2_ and a simultaneous increase in PM_2.5_. The contrast between the pattern of change of NO_2_ and PM_2.5_ following state-level emergency declarations suggests that dominating sources of these two pollutants are different in those states. It is also noticeable from Fig. 6 that, for these 13 states the changes in PM_2.5_ are not statistically significant (Table S2-b) and in 3 states (CO, GA, WA), the NO_2_ changes are not significant either (Table S2-a). We see more states with statistically significant changes in NO_2_, than PM_2.5_, following state-level emergency declarations.

### State-level factors may explain the heterogeneity in air pollution declines across states

To ascertain which state-level factors might explain the heterogeneity in the extent to which the air pollution declined across states, we fit a weighted multivariable linear regression model with the estimated ${\Delta }_{j}$ (for each pollutant separately) for each state as the dependent variable, and geography, population density and sources of emission as predictors accounting for the main effect and their corresponding two-factor interactions. For the PM_2.5_ pollutant model, all the proportions of annual emissions from a state’s stationary (e.g., industrial processes), mobile (e.g., road and air traffic), and fire sources (e.g., agricultural field burning) (Table S5, from 2014) are not statistically significant and have negative associations with the change in PM_2.5_ concentration (Table S6). In contrast, the proportion of annual emissions from mobile sources and stationary sources were statistically significantly associated with the change in NO_2_ concentration (Table S6).

## Discussion

Following the declaration of a state of emergency, we found that NO_2_ concentrations showed a statistically significant decline in 34 of the 36 states included in this analysis. In contrast, PM_2.5_ concentrations declined in only 16 of 48 states included in this analysis. These 16 states are in the Northeast and on the West Coast. Furthermore, as expected, we found that the proportion of a state’s annual emissions from mobile sources and stationary sources are statistically significant factors in NO_2_ changes in response to the state emergency declaration. For PM_2.5_ reductions, all three sources—mobile, stationary, and fire—were not statistically significant predictors and have negative associations with the changes in PM_2.5_ concentrations. We concluded that state of emergency declarations implemented in response to the COVID-19 pandemic predominantly affected mobile sources (e.g., cancelled flights and reduced traffic)^65^ and stationary sources and led to a decline in NO_2_. However, because the major sources of PM_2.5_ are stationary (e.g., industrial fuel combustion), these were less affected by state-level emergency declarations (Table S5).

SARIMA models have some advantageous features compared to other statistical approaches. Recent studies^22,23,29,36^ have used t-tests^22^, a robust difference approach^23^, linear regression^36^, and synthetic control methods^66^ to study the changes in US air pollution attributable to the COVID-19 shutdown. Bekbulat et al. used temporal correction in their robust differences approach^23^ (not peer-reviewed on August 03, 2020) and Venter et al. included meteorological factors in their regression^36^. The latter was a global study of air pollution changes during the pandemic, which found that a decline in NO_2_ in the United States occurred on a national level. However, these methods do not directly incorporate the correlations between observed pollutant concentrations, and trends and seasonality in the data. We accounted for both factors using SARIMA models. Any contribution to the data from generally decreasing air pollution trends and weather seasonality must be removed to best estimate the effect of pandemic-related extreme measures on air pollution. By further combining SARIMA with bootstrapping, we were able to quantify the uncertainty in the estimated mean predictions.

We note that our counterfactual predictions of pollutant concentrations assume that the trend and seasonality during the last five years (i.e., the training period for the model) persisted during the prediction period (January 1, 2020, to April 23, 2020). Another assumption was that the relationship between meteorological variables used in the SARIMA model (temperature, humidity, and precipitation) and the pollutant concentrations were the same in both the training and prediction periods^67,68^. While in California for NO_2_ (see Figures S1 to S5) we see smaller differences between predicted and observed concentrations before the state of emergency, in some states this was not the case, and we see differences between the predicted and observed for the entire January 2020—April 2020 period. We would not expect the model’s predictive capability to affect the estimation of the pollutant concentrations before and after the state intervention differently. Therefore, where we observed significant deviations from the predicted concentrations following the state intervention, we can be confident that it is due to the intervention and not due to the model’s predictive capability. Additionally, we fit an additional SARIMA model to predict the same prediction period for the previous year of 2019 (January 1, 2019, to April 23, 2019). In comparing the behaviour of the APEs, we find that APEs are higher for 2020 compared to 2019, except for a few states, for each of the pollutant models.

In contrast to other studies (see for example^22^), we did not a priori divide our data into pre- and post‒COVID-19 periods. We used January 1, 2015, to December 31, 2019 as historical data and then used the SARIMA model to predict the counterfactual pollutant levels during the 16-week period from January 1, 2020 to April 23, 2020, under the hypothesis that neither the pandemic nor the state emergency declaration occurred. In other words, first we predict air pollution levels for the whole study period of 16 weeks. We then looked a posteriori to determine if the NO_2_ or PM_2.5_ declines coincided with state-level emergency declarations (see Figures S1-S5 for example of NO_2_ in California).

By identifying the maximum decline in the median pollutant concentrations following state-level emergency declaration, we found that the extreme measures taken during the pandemic led to a change of PM_2.5_ of up to 3.4 µg/m^3^ (in California) and a change of NO_2_ of up to 11.6 ppb (in Nevada). These weekly-averaged values represent a substantial fraction of the annual mean NAAQS values of 12 µg/m^3^ and 53 ppb, respectively. Based on the national regression model, there is significant potential to reduce NO_2_ concentrations by reducing mobile and stationary sources of NO_2_ emissions, provided the same level of change can be sustained throughout the annual cycle. But these associations were not seen in the PM_2.5_ regression model. In Table 1, we summarized the published evidence from similar studies in the US. For example, Berman el al 2020, examines all the counties in the US for both PM_2.5_ and NO_2_. They found a 25.5% reduction (4.8 ppb) in NO_2_ during the COVID-19 period and a 11.3% statistically significant reduction (0.7 μg/m^3^) of PM_2.**5**_ in counties from states that instituted early non-essential business closures^22^. However, the statistical analysis of this study relies on t-tests and does not account for confounding or residual autocorrelation. Overall, among studies summarized in Table 1, there is consistent evidence of a decline of NO_2_ for most of the locations^22,24–26^, whereas the evidence of declines in PM_2.5_ is weaker (see for example^26,28^). In addition, one relevant pre-print study found that PM_2.5_ concentrations during lockdown are 10% (0.54 μg/m^3^) higher than expected post-covid, but 11% (0.73 μg/m^3^) lower than pre-covid, with 31% decrease in NO_2_ levels in 3 major cities^23^. Another relevant pre-print study found a nationwide average increase of 1.36 μg/m^3^ in PM_2.5_ following official lockdown orders^66^.

With respect to studies outside the US, a recent study investigated the effect of lockdown in urban China, using difference-in-difference approach; they found a decline of 14 µg/m^3^ in locked-down cities compared to cities that did not implement a lockdown^69^. The cities in that study had baseline PM_2.5_ concentrations four times higher than the safe limits set by the World Health Organization, which may have been partly responsible for a larger decline after lockdown compared to what we observed in the United States. Another study used baseline regression to estimate the impact of lockdown on 44 cities in Northern China and found a 5.93% decrease in PM_2.5_ and a 24.67% decrease in NO_2_ concentrations during lockdown^40^. Others have used paired t-tests and the autoregressive moving average (ARMA) model to quantify the impact of the COVID-19 lockdown in 41 cities in India on pollution levels, and found a 19% decrease in NO_2_ compared to the same period in 2019^45^.

Our study results support the effectiveness of state-level actions to reduce ambient levels of PM_2.5_ and NO_2_, and specifically, that restrictions on stationary and mobile sources of air pollution could decrease NO_2_ emissions even further in states where mobile sources constitute a larger proportion of annual emissions. In contrast, PM_2.5_ concentration reduction may not be as easily achieved through these sources alone. In states where changes in PM_2.5_ and NO_2_ exhibited opposite trends (one increased while the other decreased), lowering the emission of NO_2_, by decreasing mobile source emissions for example, may not necessarily decrease PM_2.5_ concentrations.

### Study limitations

The models were fit separately for PM_2.5_ and NO_2_ and we did not account for correlation between the two pollutants. We relied on state-level concentration averages and the 2014 emissions inventory. While our study would benefit greatly from a more recent emissions inventory (or spatial emissions estimates during the interventions), to our knowledge, such data is not currently available publicly. With respect to fire emissions, we note that although we only have data from 2014, regions with higher areas burned in 2014 have larger propensity to have higher areas burned in 2020^70^. Trading finer spatial resolution in the monitoring data—not averaging to the state level—may reveal important sub-state variability in lockdown impacts. Monitor data was obtained from EPA AirNow and has not undergone quality control by the EPA. We didn’t remove outlier observations, however we averaged hourly measurements by day and by state, which would have minimized the impact of outliers. Our approach also does not consider the spatial correlations between pollutant concentrations, which may help explain concentration changes in non-local pollutants such as PM_2.5_. Wind speed was not included in the SARIMA model, adjusting for wind speed could have improved the predictions even more. Finally, data were available for 36 states for NO_2_ and 48 states for PM_2.5_, which limited the number of observations in the weighted regression model. Finally, even though we have accounted for the interactions between the predictors in the weighted least squares regression models, we need to consider adjusting for other potential predictors which could improve the prediction.

## Supplementary Information

Supplementary Information.
