# Supplementary material for: Counterfactual time series analysis of short-term change in air pollution following the COVID-19 state of emergency in the United States
Source: Sci Rep. 2021 Dec 7;11:23517. doi: 10.1038/s41598-021-02776-0 (PMC8651777; doi:10.1038/s41598-021-02776-0)
Supplement: Supplementary file 1 — Supplementary Information. [file 41598_2021_2776_MOESM1_ESM.docx]

**SUPPLEMENTARY MATERIALS**

**Counterfactual time series analysis of short-term change in air pollution following the COVID-19 state of emergency in the United States**

Tanujit Dey^+1^, Pooja Tyagi^+2^, Benjamin Sabath^2^, Leila Kamareddine^2^, Lucas Henneman^4^, Danielle Braun^2,5^, and Francesca Dominici^2^*

^1^Center for Surgery and Public Health, Department of Surgery, Brigham and Women’s Hospital, Harvard Medical School

^2^Department of Biostatistics, Harvard T.H. Chan School of Public Health, 677 Huntington Ave, Boston, MA 02115, United States

^3^Faculty of Arts and Sciences, Research Computing, Harvard University, 38 Oxford Street, Cambridge, MA 02138, United States

^4^Department of Civil, Environmental, and Infrastructure Engineering, George Mason University, 4400 University Drive Fairfax, Virginia 22030, United States

^5^ Department of Data Science, Dana-Farber Cancer Institute, 450 Brookline Ave, Boston, MA 02215, United States

*Corresponding Author*:* [fdominic@hsph.harvard.edu](mailto:fdominic@hsph.harvard.edu)

**Table S1.** Publicly available data sources used in the analysis.

| **Data** | **Source** |
| --- | --- |
| Daily monitor data for PM_2.5_ and NO_2_ emissions from January 1, 2015 to August 31, 2019 | US Environmental Protection Agency Air Quality System (53) |
| County-level daily data for PM_2.5_ and NO_2_ emissions after August 31, 2019 | EPA AirNow (54) |
| Daily temperature, humidity, and precipitation for all states | University of Idaho’s GRIDMET project (55) aggregated to county from 4km x 4km rasters |
| State-level source emissions totals | National emissions inventory (56) |
| State population density and Regions | 2010 Census data (57,58) |
| Timing of state interventions | COVID-19 US state policy database (59) |

**SARIMA analysis for NO_2­_ concentrations in California**

This section describes the step-by-step time series analysis for counterfactual prediction of NO_2_ in California to illustrate the details of our approach. Figure S1 (a) shows raw data for daily average NO_2_ levels for five years (January 1, 2015 to December 12, 2019). For our model, we estimated weekly averages resulting in 261 data points for the five-year period, one time point for each week (Figure S1 (b)).

**Figure S1.** (a) Time series data for daily average NO_2_ levels for California for a five-year period. (b) Weekly averages of data in (a) used for training the SARIMA model.


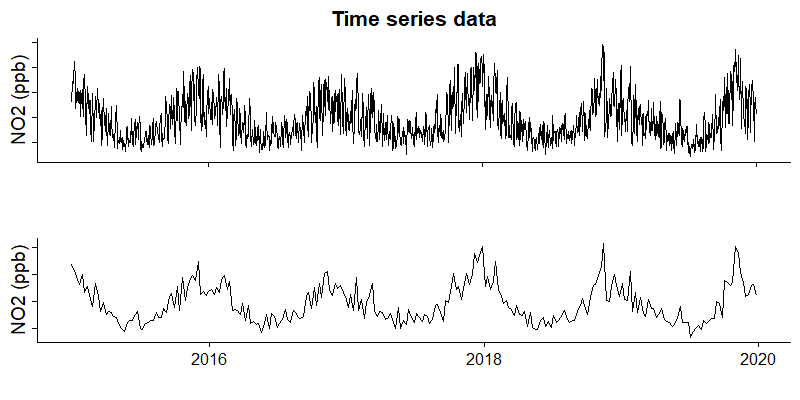


First, we created 1,000 bootstrapped time series from the weekly averaged training data using Box-Cox and Loess based decomposition.(39) Figure S2 shows a plot of 95% CI of these bootstraps.

Next, we fit a SARIMA model to each of these bootstrapped time series, adjusting for meteorological factors, namely temperature, precipitation, and humidity. The average of the fitted values of the model is shown in Figure S3 along with 95% CI. We found that the model fits the data well with a mean absolute scaled error (MASE) = 0.8. Subsequently, we used the 1,000 fitted models to make counterfactual forecasts of weekly averages for 16 weeks in 2020 (January to April). Figure S4 shows the average predictions from the 1,000 models and the corresponding 95% CI. A comparison of the forecasts of the observed data shows that the NO_2_ levels begin to deviate from the predictions at about 10 weeks.

**Figure S2.** Time series data of weekly averages of NO_2_ levels in California (black line) with the confidence intervals of the 1,000 generated bootstraps (shaded area).


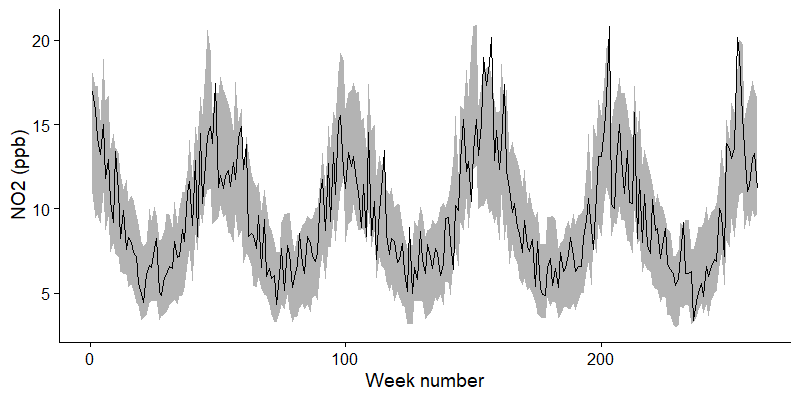


**Figure S3.** Average of the fitted NO_2_ values in California from 1,000 SARIMA models (red), corresponding 95% CI (shaded area), and observed data (black).


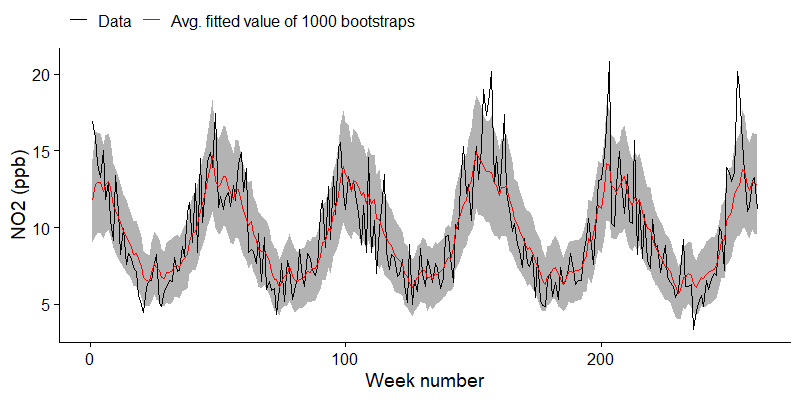


Finally, we calculated the difference between the average of the fitted predictions and the observed data (Figure S5). We found that the NO_2_ levels start deviating from our forecasts at the same time as the state of emergency was declared in California. The analysis described above was performed for each state and for PM_2.5_ and NO_2_.


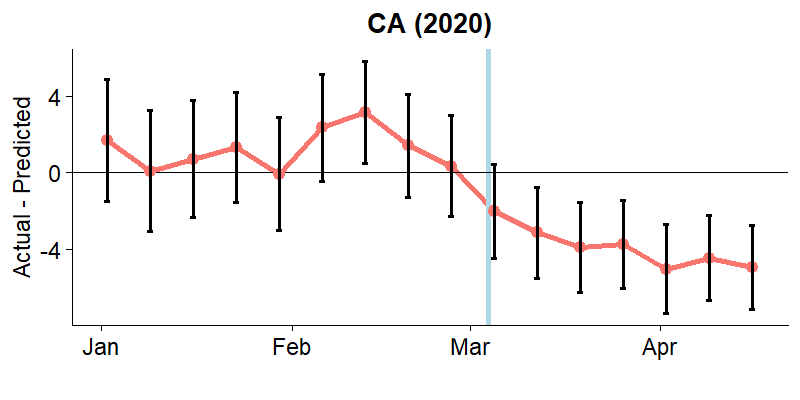


**Figure S5.** The weekly deviations between actual and predicted NO_2­_ levels (ppb) for 16 weeks in 2020 in California. The blue line corresponds to the day of declaration of a state of emergency in California.

**Figure S4.** Average of predictions from 1,000 SARIMA models for 16 weeks in 2020 (red) for NO_2_ levels in California, corresponding 95% CI (shaded area), and observed data (black).


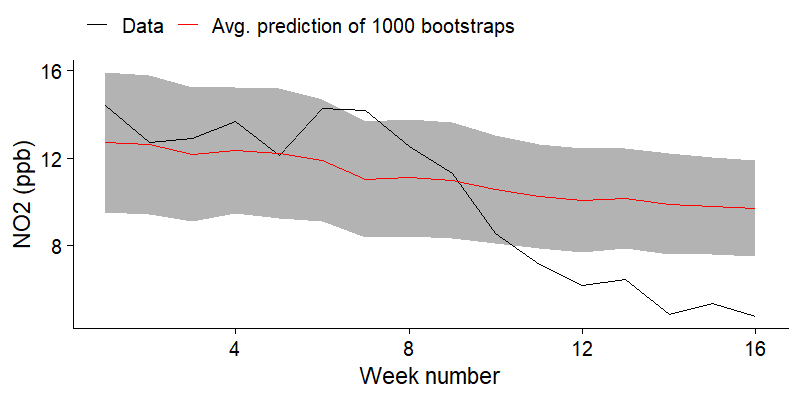


**Model Evaluation**

Figure S6 shows the MASE distribution (65) for all fitted models.

**Figure S6.** Mean absolute scaled error (MASE) for fitted models for all states for (a) NO_2_ and (b) PM_2.5_.


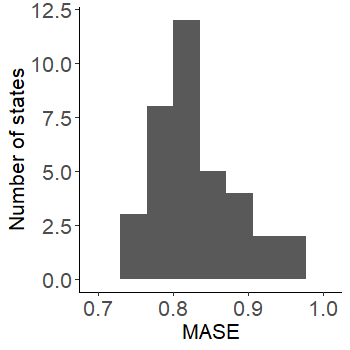

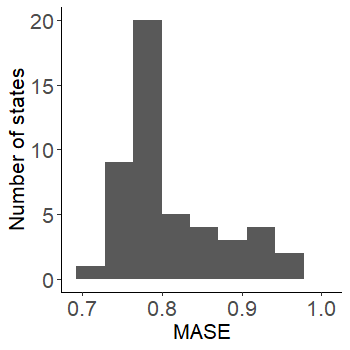


**Tests for statistical significance in decrease in pollutant levels**

**Table S2-a.** P-values obtained from Mann-Whitney test comparing the deviation from predicted values before versus after the declaration of a state of emergency in each state for NO_2_.

| State | P-value |  | State | P-value |
| --- | --- | --- | --- | --- |
| AZ | < 0.001 |  | **KY** | 0.005 |
| CA | < 0.001 |  | **MO** | 0.007 |
| CT | < 0.001 |  | **OK** | 0.007 |
| MA | < 0.001 |  | **WI** | 0.008 |
| NV | < 0.001 |  | **IN** | 0.011 |
| NM | < 0.001 |  | **IA** | 0.011 |
| PA | < 0.001 |  | **SC** | 0.019 |
| RI | < 0.001 |  | **MI** | 0.021 |
| UT | < 0.001 |  | **OR** | 0.021 |
| VT | < 0.001 |  | **KS** | 0.028 |
| VA | < 0.001 |  | **OH** | 0.028 |
| NJ | 0.001 |  | **MN** | 0.057 |
| MS | 0.002 |  | **WA** | 0.071 |
| NC | 0.002 |  | **CO** | 0.074 |
| ND | 0.002 |  | **GA** | 0.09 |
| ID | 0.003 |  | **WY** | 0.09 |
| ME | 0.004 |  | **MT** | 0.356 |
| MD | 0.004 |  | **FL** | 0.604 |

**Table S2-b.** P-values obtained from Mann-Whitney test comparing the deviation from predicted values before versus after the declaration of a state of emergency in each state for PM_2.5_.

| State | P-value |  | State | P-value |
| --- | --- | --- | --- | --- |
| AL | 0.84 |  | **NE** | 0.889 |
| AZ | 0.005 |  | **NV** | < 0.001 |
| AR | 0.719 |  | **NH** | 0.007 |
| CA | < 0.001 |  | **NJ** | 0.028 |
| CO | 0.644 |  | **NM** | 0.008 |
| CT | 0.004 |  | **NY** | < 0.001 |
| DE | 0.811 |  | **NC** | 0.964 |
| FL | 0.964 |  | **ND** | 0.413 |
| GA | 0.955 |  | **OH** | 0.157 |
| ID | 0.019 |  | **OK** | 0.927 |
| IL | 0.604 |  | **OR** | 0.028 |
| IN | 0.682 |  | **PA** | 0.011 |
| IA | 0.318 |  | **RI** | < 0.001 |
| KS | 0.868 |  | **SC** | 0.889 |
| KY | 0.816 |  | **SD** | 0.413 |
| LA | 0.995 |  | **TN** | 0.786 |
| ME | 0.003 |  | **TX** | 1 |
| MD | 0.235 |  | **UT** | < 0.001 |
| MA | 0.001 |  | **VT** | 0.004 |
| MI | 0.816 |  | **VA** | 0.682 |
| MN | 0.255 |  | **WA** | 0.459 |
| MS | 0.996 |  | **WV** | 0.457 |
| MO | 0.371 |  | **WI** | 0.754 |
| MT | 0.246 |  | **WY** | 0.111 |

**Table S3.** Changes in NO_2_ concentration (Δ) by state.

| State | NO_2_ change (ppb) |  | State | NO_2_ change (ppb) |
| --- | --- | --- | --- | --- |
| AZ | 5.994647 |  | **MT** | 0.214428 |
| CA | 5.399636 |  | **NC** | 1.944749 |
| CO | 1.621158 |  | **ND** | 0.749082 |
| CT | 5.215251 |  | **NJ** | 5.738134 |
| FL | -0.55993 |  | **NM** | 3.665656 |
| GA | 1.899548 |  | **NV** | 11.60348 |
| IA | 0.849 |  | **OH** | 2.042254 |
| ID | 3.996095 |  | **OK** | 2.928278 |
| IN | 2.06196 |  | **OR** | 1.799317 |
| KS | 1.230293 |  | **PA** | 6.381725 |
| KY | 3.670385 |  | **RI** | 7.177902 |
| MA | 5.219282 |  | **SC** | 0.813475 |
| MD | 2.583846 |  | **UT** | 5.140808 |
| ME | 3.073439 |  | **VA** | 2.368074 |
| MI | 2.389468 |  | **VT** | 4.511339 |
| MN | 1.893442 |  | **WA** | 2.124366 |
| MO | 2.212506 |  | **WI** | 2.366271 |
| MS | 2.256004 |  | **WY** | 0.281806 |

| State | PM_2.5_ change (µg/m^3^) |  | State | PM_2.5_ change (µg/m^3^) |
| --- | --- | --- | --- | --- |
| AL | -1.34 |  | **NC** | -1.01 |
| AR | -0.24 |  | **ND** | 0.24 |
| AZ | 2.44 |  | **NE** | -2.00 |
| CA | 3.37 |  | **NH** | 1.15 |
| CO | -0.44 |  | **NJ** | 1.41 |
| CT | 1.90 |  | **NM** | 1.05 |
| DE | -0.58 |  | **NV** | 2.72 |
| FL | -0.73 |  | **NY** | 2.26 |
| GA | -1.62 |  | **OH** | 0.68 |
| IA | 0.93 |  | **OK** | -2.04 |
| ID | 0.92 |  | **OR** | 1.61 |
| IL | -0.14 |  | **PA** | 1.48 |
| IN | -0.23 |  | **RI** | 1.80 |
| KS | -1.15 |  | **SC** | -0.31 |
| KY | -0.18 |  | **SD** | -0.44 |
| LA | -1.44 |  | **TN** | -0.30 |
| MA | 1.23 |  | **TX** | -2.28 |
| MD | 0.31 |  | **UT** | 1.58 |
| ME | 1.23 |  | **VA** | -0.41 |
| MI | -0.72 |  | **VT** | 1.74 |
| MN | 0.95 |  | **WA** | -0.11 |
| MO | 0.24 |  | **WI** | -0.32 |
| MS | -1.95 |  | **WV** | 0.38 |
| MT | 0.49 |  | **WY** | 0.32 |

**Table S4.** Changes in PM_2.5_ concentration (Δ) by state.


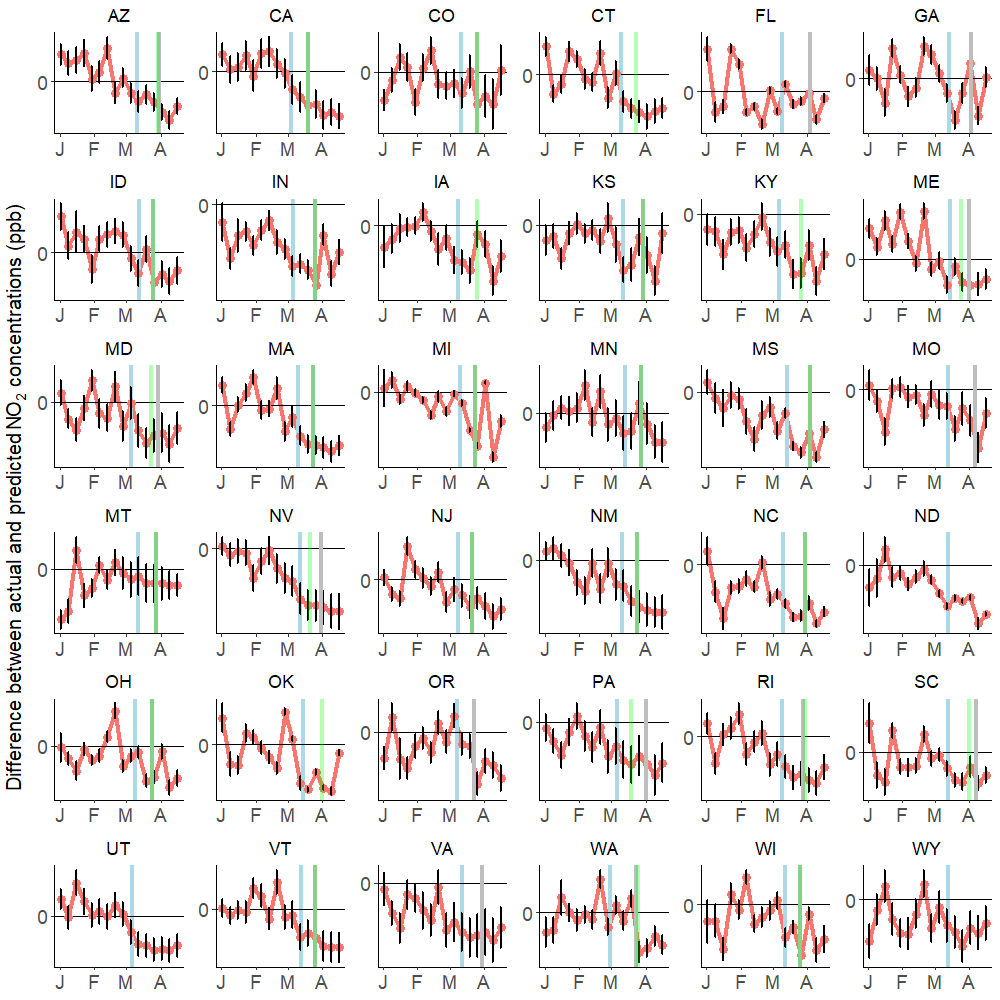


**Figure S7.** Weekly deviations between observed NO_2_ concentrations and counterfactual predictions (e.g., absent the pandemic) for each state. The predictions were made for 16 weeks from January 1 to April 23, 2020. The blue, green, and grey vertical lines mark the dates corresponding to the declaration of a state of emergency, non-essential business closures, and shelter-in-place/stay-at-home orders in each state.


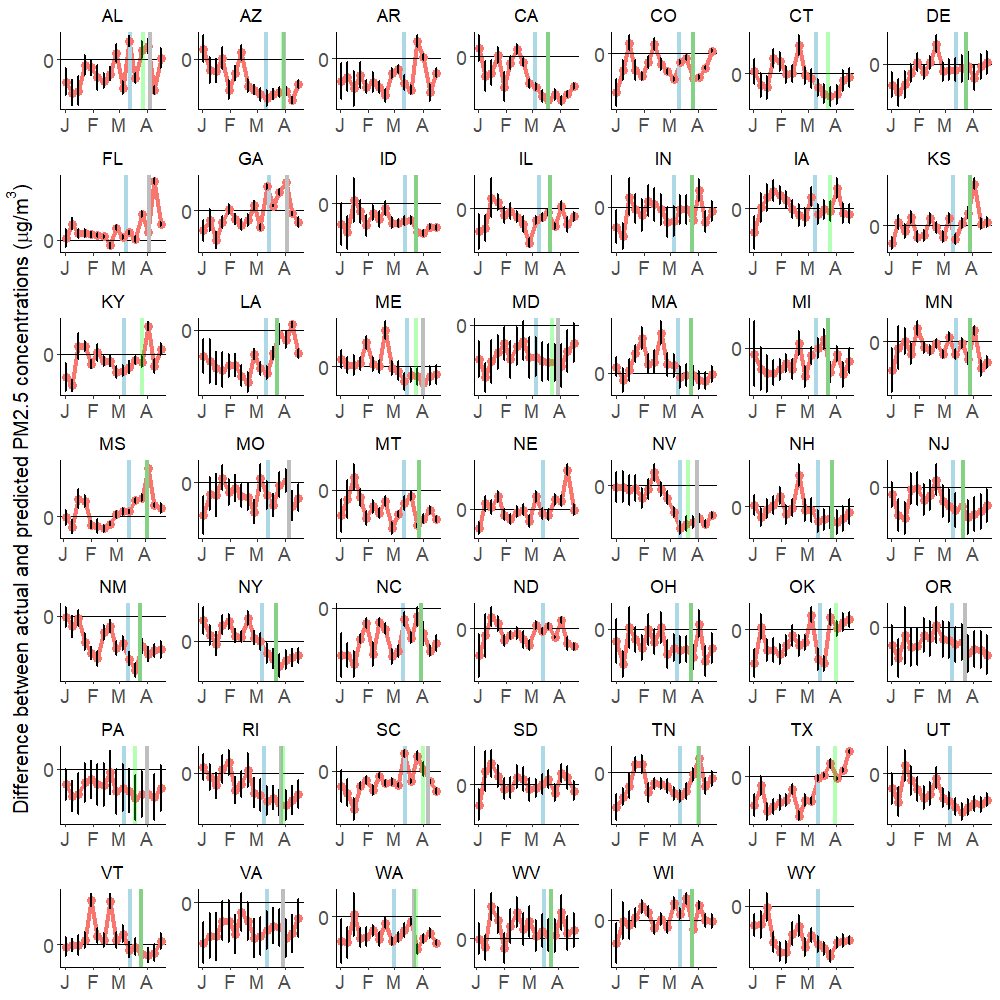


**Figure S8.** Weekly deviations between observed PM_2.5_ concentrations and counterfactual predictions (e.g., absent the pandemic) for each state. The predictions were made for 16 weeks from January 1 to April 23, 2020. The blue, green, and grey vertical lines mark the dates corresponding to the declaration of a state of emergency, non-essential business closures, and shelter-in-place/stay-at-home orders in each state.


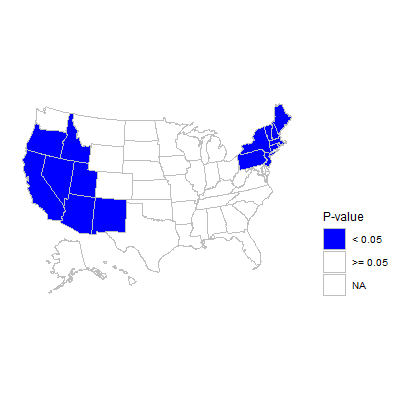


**Figure S9.** States with a statistically significant difference (P < 0.05) in deviations from predicted values before versus after the declaration of a state of emergency are highlighted in blue. (map created using R version 3.6.2)

**
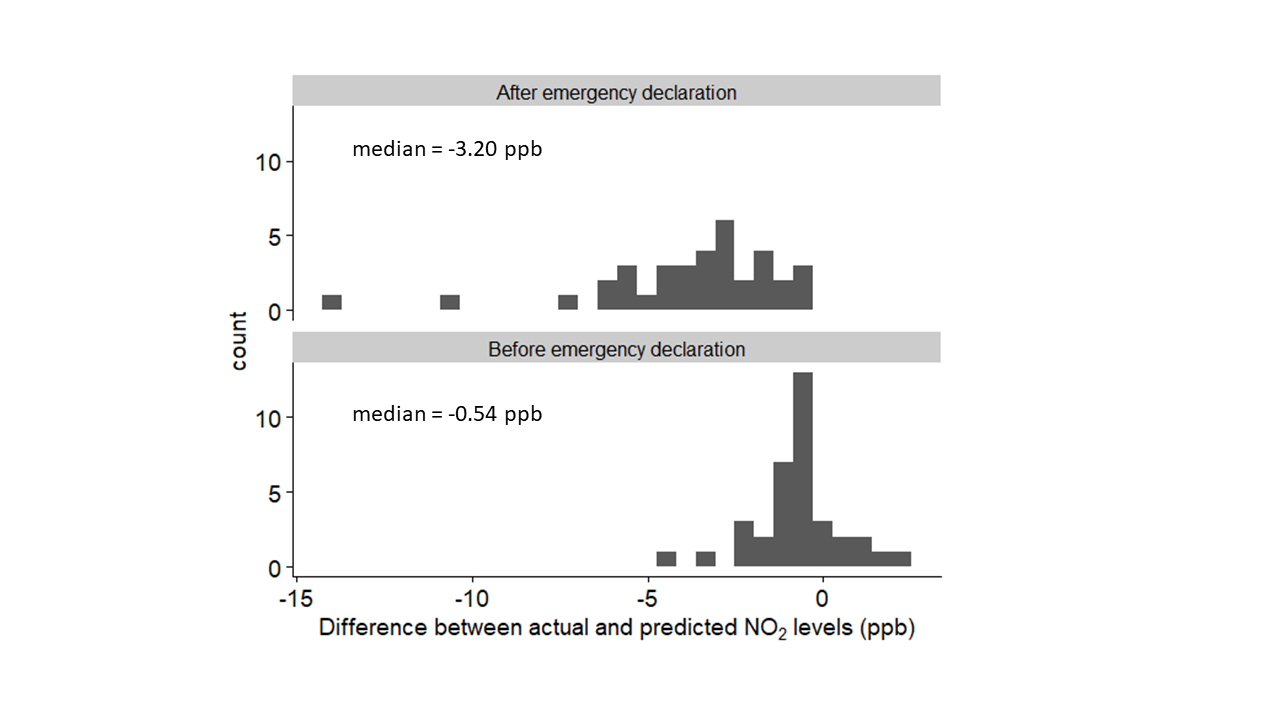
**

**Figure S10:** Difference between actual and predicted NO_2_ levels in all states after the state of emergency declarations (top) and before the state of emergency declarations (bottom).

**
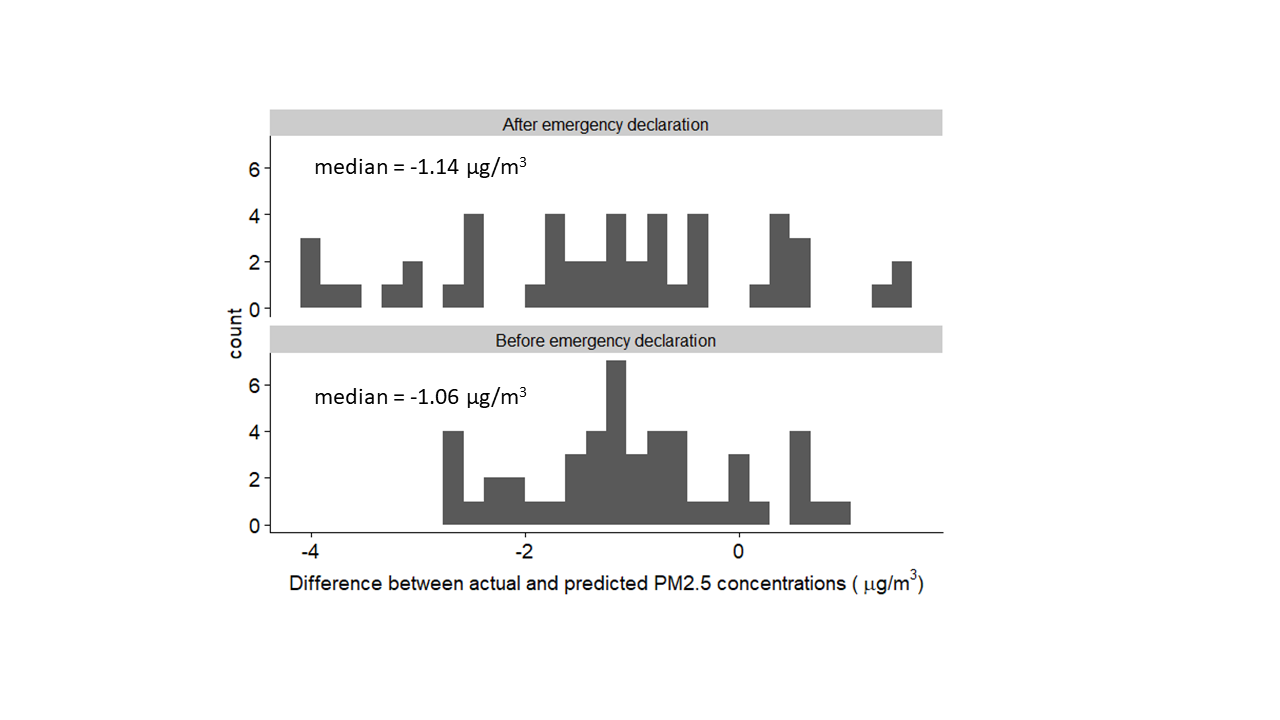
**

**Figure S11**: Difference between actual and predicted PM_2.5_ levels in all states after the state of emergency declarations (top) and before the state of emergency declarations (bottom).

**National level pollutant emissions**

| **Table S5.** Annual PM_2.5_ and NO_2_ emissions (in tons) by emission source in the continental United States in 2014.(35) | | |
| --- | --- | --- |
| **Sources** | **Emission (Tons)** | |
|  | **NO_2_** | **PM_2.5_** |
| **Stationary** | 4,649,676 (34.8%) | 3,292,221 (61.4%) |
| **Mobile** | 7,513,708 (56.2%) | 336,554 (6.3%) |
| **Fire** | 291,213 (2.2%) | 1,730,360 (32.3%) |
| **Biogenics** | 903,124 (6.8%) | 0 (0.0%) |

**Table S6.** Estimated coefficients, 95% confidence intervals (CI) and P-values for variables included in the weighted multivariable linear regression analysis. The categorical variable *Region* refers to the geographical regions of the United States and has four categories as defined by the US Census, *Region 1: Northeast*, *Region 2: Midwest*, *Region 3: South* and *Region 4: West*. The corresponding coefficients are reported using *Region 1* as reference.

|  | **NO_2_ change** | | | **PM_2.5_ change** | | |
| --- | --- | --- | --- | --- | --- | --- |
| *Predictors* | *Estimates* | *95% CI* | *P-value* | *Estimates* | *95% CI* | *P-value* |
| (Intercept) | -8.47 | -26.61 – 9.66 | 0.322 | -1.55 | -7.51 – 4.42 | 0.596 |
| Fire Sources | 3.65 | -10.10 – 17.39 | 0.567 | -5.15 | -14.58 – 4.29 | 0.270 |
| Mobile Sources | 13.14 | 0.76 – 25.52 | **0.040** | -1.55 | -4.32 – 1.23 | 0.260 |
| Stationary Sources | 18.42 | 5.32 – 31.53 | **0.011** | -1.44 | -3.96 – 1.09 | 0.250 |
| Population density | 0.01 | -0.00 – 0.02 | 0.148 | 0.01 | -0.00 – 0.01 | 0.201 |
| Region 2 | 8.91 | -8.04 – 25.86 | 0.269 | 0.94 | -4.39 – 6.26 | 0.719 |
| Region 3 | 10.74 | -8.06 – 29.55 | 0.232 | -0.03 | -5.46 – 5.39 | 0.990 |
| Region 4 | 15.04 | -5.65 – 35.73 | 0.136 | 2.93 | -3.52 – 9.38 | 0.356 |
| Fire Sources * Mobile Sources | -0.45 | -2.13 – 1.24 | 0.568 | -0.20 | -1.28 – 0.89 | 0.713 |
| Fire Sources * Stationary Sources | 2.30 | 0.66 – 3.94 | **0.011** | -0.37 | -1.45 – 0.70 | 0.478 |
| Fire Sources * Population density | -0.01 | -0.06 – 0.03 | 0.598 | -0.01 | -0.03 – 0.01 | 0.405 |
| Fire Sources * Region 2 | -2.74 | -14.05 – 8.57 | 0.601 | 6.63 | -1.84 – 15.10 | 0.119 |
| Fire Sources * Region 3 | 1.26 | -13.42 – 15.95 | 0.852 | 8.94 | 0.86 – 17.02 | **0.032** |
| Fire Sources * Region 4 | 2.03 | -12.72 – 16.78 | 0.765 | 4.58 | -5.60 – 14.76 | 0.361 |
| Mobile Sources * Stationary Sources | 1.21 | -0.60 – 3.02 | 0.167 | 0.02 | -0.47 – 0.51 | 0.945 |
| Mobile Sources * Population density | -0.01 | -0.05 – 0.02 | 0.379 | -0.01 | -0.03 – 0.01 | 0.259 |
| Mobile Sources * Region 2 | -11.99 | -27.19 – 3.20 | 0.109 | 1.79 | -1.25 – 4.82 | 0.235 |
| Mobile Sources * Region 3 | -8.43 | -32.16 – 15.30 | 0.447 | 5.89 | 1.74 – 10.04 | **0.007** |
| Mobile Sources * Region 4 | -7.42 | -18.77 – 3.93 | 0.176 | 2.58 | -0.33 – 5.49 | 0.079 |
| Stationary Sources * Population density | -0.02 | -0.05 – 0.01 | 0.109 | -0.01 | -0.03 – 0.01 | 0.254 |
| Stationary Sources * Region 2 | -14.22 | -28.06 – -0.39 | **0.045** | 2.22 | -0.62 – 5.05 | 0.119 |
| Stationary Sources * Region 3 | -11.06 | -33.93 – 11.81 | 0.307 | 5.59 | 1.86 – 9.31 | **0.005** |
| Stationary Sources * Region 4 | -13.98 | -25.96 – -1.99 | **0.027** | 1.10 | -1.59 – 3.80 | 0.405 |
| Population density * Region 2 | 0.00 | -0.01 – 0.02 | 0.475 | 0.00 | -0.01 – 0.01 | 0.705 |
| Population density * Region 3 | -0.01 | -0.01 – 0.00 | 0.056 | -0.00 | -0.01 – 0.00 | 0.296 |
| Population density * Region 4 | -0.00 | -0.05 – 0.05 | 0.958 | -0.01 | -0.02 – 0.01 | 0.329 |
| R^2^ / R^2^ adjusted | 1.000 / 1.000 | | | 0.986 / 0.970 | | |
